# Supplementary material for: Assessing Multiple Evidence Streams to Decide on Confidence for Identification of Post-Translational Modifications, within and Across Data Sets
Source: J Proteome Res. 2023 Apr 26;22(6):1828–42. doi: 10.1021/acs.jproteome.2c00823 (PMC10243108; doi:10.1021/acs.jproteome.2c00823)
Supplement: Supplementary file 1 — pr2c00823_si_001.pdf [file pr2c00823_si_001.pdf]

Assessing multiple evidence streams to decide on confidence for identification of post-translational modifications, within and across data sets

Oscar M Camacho, Kerry Ramsbottom, Andrew Collins and Andrew R Jones

Institute of Systems, Molecular and Integrative Biology, University of Liverpool, UK, L69 7ZB

SUPPLEMENTARY INFORMATION

Table of contents

- Supp Table 1. Database search parameters for PTM identification in TPP.
- Supp Table 2. Database search parameters for PTM identification in PD.
- Supp Figure 1. Number of PSMs matching each type of phosphor site for data analysed using PD.
- Supp Figure 2. FLR curves for unadjusted and binomial adjusted PD results, PSM level.
- Supp Table 3. PSM-site counts for unadjusted and binomial adjusted PD results.
- Supp Figure 3. FLR curves for unadjusted, binomial adjusted and product adjusted PD results. Unadjusted and binomial adjusted 3 collapsing approaches.
- Supp Table 4. Number of phosphor-sites at peptidoform level for unadjusted, binomial adjusted and product adjusted PD results. Unadjusted and binomial adjusted 3 collapsing approaches.

Supp Table 1. Parameters used for peptide identification and PTM site localisation using TPP.

Data set ProteomeXchange Unique ID	Peptide Mass Tolerance (ppm)	Fragment Bin Tolerance (Da)	Digest Mode	Max Missed Cleavages	Fixed Mods	Variable Mods	Max Phospho sites
PXD000138	5	0.02	Tryptic	4	Carbamidomethylation (C)	Oxidation (M), Phospho (STYA), N-terminal acetylation, Ammonia loss (QC), Pyro-Glu (EQ on the N-terminus), Deamination (NQ)	5
PXD007058	20	0.02	Tryptic	4	Carbamidomethylation (C)	Oxidation (MWP), Phospho (STYA), Pyrophospho (STY)¶, N-terminal acetylation, Ammonia loss (QC), Pyro-Glu (EQ on the N-terminus), Deamination (NQ)	5
PXD008355	7	0.02	Tryptic	2	Carbamidomethylation (C)	Oxidation (M), Phospho (STYA), N-terminal acetylation, Ammonia loss (QC), Pyro-Glu (EQ on the N-terminus), Deamination (NQ)	5
PXD000612	7	0.02	Tryptic	2	Carbamidomethylation (C)	Oxidation (M), Phospho (STYA), N-terminal acetylation, Ammonia loss (QC), Pyro-Glu (EQ on the N-terminus), Deamination (NQ)	5
PXD000923	50	0.02	Tryptic	2	Carbamidomethylation (C)	Oxidation(MW) N-terminal acetylation, ammonia loss (QC), pyro-glu (E), deamination (NQ) and phospho (STYA)	5
PXD002222	20	0.02	Tryptic	2	Carbamidomethylation (C)	Oxidation(MW) N-terminal acetylation ammonia loss (QC), pyro-glu (E), deamination (NQ) and phospho(STYA)	5
PXD002756	20	1.0005	Tryptic	2	Carbamidomethylation (C)	Oxidation(MW) N-terminal acetylation ammonia loss (QC), pyro-glu (E), deamination (NQ) and phospho (STYA)	5
PXD004705	20	0.02	Tryptic	2	Carbamidomethylation (C)	Oxidation(MW) N-terminal acetylation ammonia loss (QC), pyro-glu (E), deamination	5

						(NQ) and phospho (STYA)	
PXD004939	20	0.02	Tryptic	2	Carbamidomethylation (C)	Oxidation(MW) N-terminal acetylation ammonia loss (QC), pyro-glu (E), deamination (NQ) and phospho (STYA)	5
PXD005241	20	1.0005	Tryptic	2	Carbamidomethylation (C)	Oxidation(MW) N-terminal acetylation ammonia loss (QC), pyro-glu (E), deamination (NQ) and phospho (STYA)	5
PXD012764	10	0.02	Tryptic	2	Carbamidomethylation (C) iTRAX8plex label	Oxidation(MW) N-terminal acetylation ammonia loss (QC), pyro-glu (E), deamination (NQ) and phosphor (STYA)	5
PXD019291	10	0.02	Tryptic	2	Carbamidomethylation (C)	Oxidation(MW) N-terminal acetylation ammonia loss (QC), pyro-glu (E), deamination (NQ) and phosphor (STYA)	5

Supp Table 2. Parameters used for peptide identification and PTM site localisation using Proteome Discoverer Mascot/ptmRS.

	Peptide Mass Tolerance	Fragment Bin Tolerance	Digest Mode	Max missed cleavages	Fixed Mods	Variable Mods	Max Phospho sites
PXD007058 (Synthetic data set)	20.0 ppm	0.02 Da	Tryptic	2	Carbamidomethylation (C)	Oxidation (MWP) Phospho (STYA) Pyrophospho (STY)	3
PXD008355 (Arabidopsis data set)	10.0 ppm	0.02 Da	Tryptic	2	Carbamidomethylation (C)	Oxidation (M) Phospho (STYA) N-terminal acetylation Ammonia loss (QC) Pyro-Glu (EQ on the Nterminus) Deamination (NQ)	3

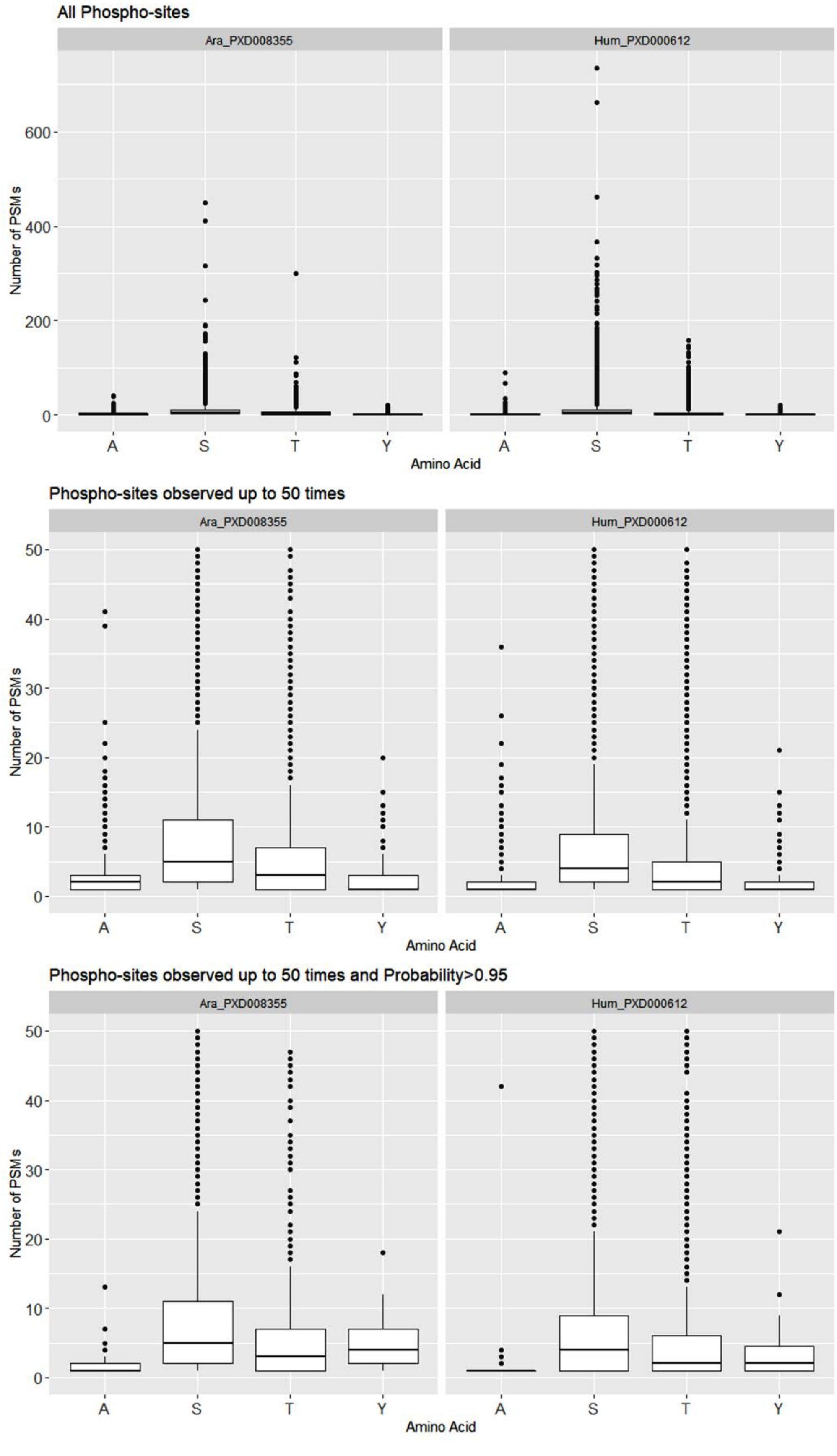

Supp Figure 1. Number of PSMs matching specific sites by type of match for Human and Arabidopsis natural data sets analysed using the PD pipeline by amino acid. Top 2 panels display all data, middle 2 panels display phosphosites observed up to 50 times and, the bottom 2 panels are those phosphosites with scores probability above 0.95 and observed up to 50 times.

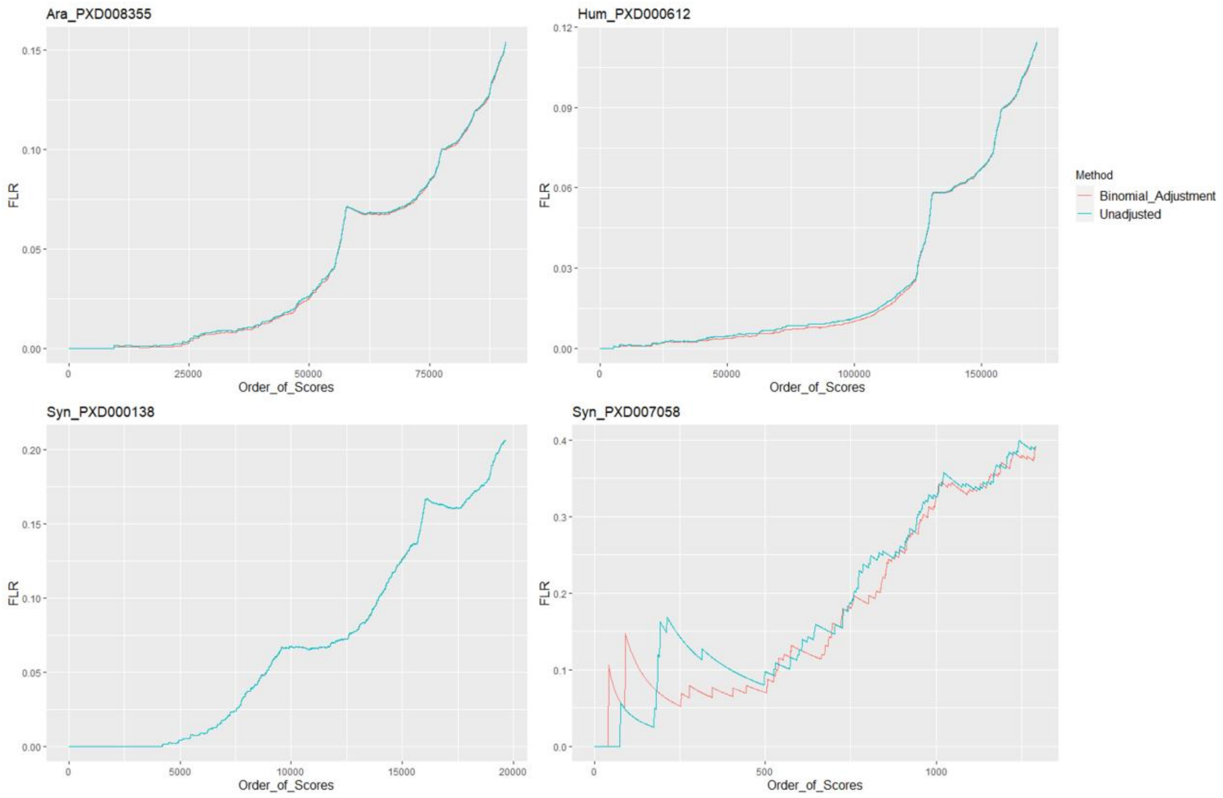

Supp Figure 2. pAla FLR at PSM-site level by ordered unadjusted scores (Blue), using the Bi-nomial_Adjustment (Red). Data sets analysed using PD pipeline.

Supp Table 3. Number of PSM-site matches for Arabidopsis, Human and two synthetic data sets analysed using the PD pipeline and thresholds at 1%, 2.5%, 5% and 10% pAla FLR based on unadjusted results and adjusted using the Product_Adjusted and Binomial Adjusted approaches. For the synthetic data sets, the Real_FLR is also displayed, calculated as the proportion of pAla PSM-site matches at each pAla FLR threshold.

	Arabidopsis PXD008355					Human PXD000612					Synthetic PXD000138									Synthetic PXD007058								
		pAla FLR					pAla FLR					pAla FLR									pAla FLR							
	Total	10%	5%	2.5%	1%	Total	10%	5%	2.5%	1%	Total	10%	5%	2.5%	1%	10%	5%	2.5%	1%	Total	10%	5%	2.5%	1%	10%	5%	2.5%	1%
	Number of observations					Number of observations					Number of observations					Real FLR				Number of observations					Real FLR			
Unadjusted	90800	77507	56105	48854	36549	171546	165414	129633	122993	93253	19641	13933	8886	7537	6260	7.0%	3.8%	2.5%	1.4%	1293	524	181	175	75	15.4%	18.2%	17.7%	9.3%
Binomial_Adjusted		78345	56149	50012	38229		165566	129622	123818	99929		13930	8884	7535	6258	7.0%	3.8%	2.4%	1.4%		537	89	41	41	13.4%	16.9%	14.6%	14.6%

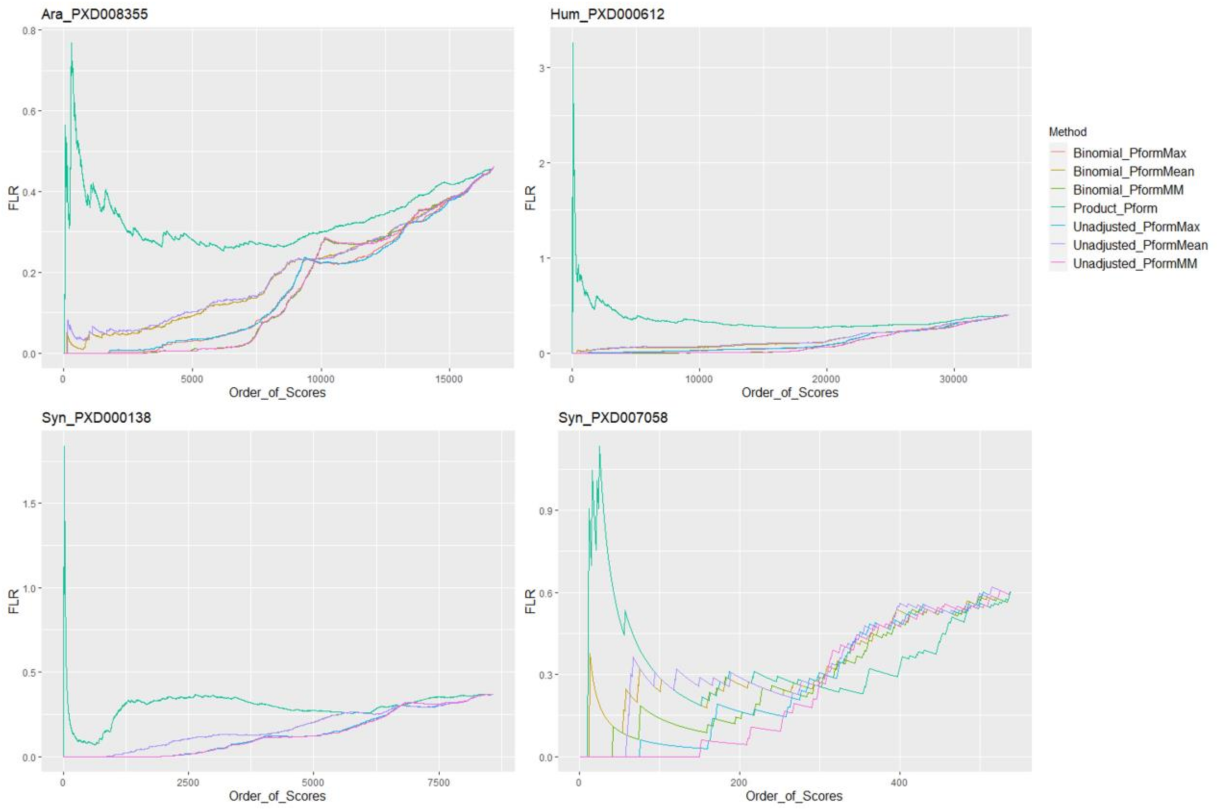

Supp Figure 3. pAla FLR at Peptidoform-site level by ordered scores for six collapsing approaches in two natural data sets and two synthetic. Data analysed using the PD pipeline.

Supp Table 4. Data sets analysed using the PD pipeline. Number of phosphosites at Peptidoform-site level for six collapsing approaches. Results are reported overall and for pAla 1%, 5% and 10% FLR thresholds. Real FLR is also displayed for the synthetic data sets.

	Arabidopsis PXD008355				Human PXD000612				Synthetic PXD000138							Synthetic PXD007058						
		pAla FLR				pAla FLR				pAla FLR							pAla FLR					
	Total	10%	5%	1%	Total	10%	5%	1%	Total	10%	5%	1%	10%	5%	1%	Total	10%	5%	1%	10%	5%	1%
	Number of observations				Number of observations				Number of observations				Real FLR			Number of observations				Real FLR		
Unadjusted_PformMax		8058	6636	3516		20821	16250	4597		3858	3186	2406	7.0%	4.3%	2.0%		164	160	75	18.9%	18.1%	9.3%
Unadjusted_PformMean		4957	1669	168		14489	3657	582		2394	1683	1030	7.3%	4.2%	2.1%		60	58	58	30.0%	27.6%	27.6%
Unadjusted_PformMM		8493	7521	5324		21521	18676	15736		3875	3248	2405	6.8%	4.4%	1.9%		251	209	150	23.0%	17.9%	17.3%
Product_Pform	16689	49	44	44	34359	5	5	5	8589	770	5	5	11.3%	0.0%	0.0%	538	10	10	10	10.0%	10.0%	10.0%
Binomial_PformMax		8051	6473	3694		20819	17570	4172		3857	3185	2405	7.0%	4.2%	1.9%		159	41	41	18.2%	14.6%	14.6%
Binomial_PformMean		5351	2916	792		16842	2945	327		2393	1682	1029	7.2%	4.2%	2.0%		54	12	12	27.8%	50.0%	50.0%
Binomial_PformMM		8458	7494	5602		21578	18700	15917		3874	3247	2404	6.8%	4.4%	1.9%		159	41	41	18.2%	14.6%	14.6%
